# The Local and Systemic Humoral Immune Response Against Homologous and Heterologous Strains of the Type 2 Porcine Reproductive and Respiratory Syndrome Virus

**DOI:** 10.3389/fimmu.2021.637613

**Published:** 2021-03-09

**Authors:** Andrew R. Kick, Amanda F. Amaral, Alba Frias-De-Diego, Lizette M. Cortes, Jonathan E. Fogle, Elisa Crisci, Glen W. Almond, Tobias Käser

**Affiliations:** Department of Population Health and Pathobiology, College of Veterinary Medicine, North Carolina State University, Raleigh, NC, United States

**Keywords:** adaptive immunity, humoral immunity, neutralizing antibodies, IgA, IgG, porcine reproductive and respiratory syndrome virus

## Abstract

The humoral immune response plays a crucial role in the combat and protection against many pathogens including the economically most important, highly prevalent, and diverse pig pathogen PRRSV – the Porcine Reproductive and Respiratory Syndrome Virus. In addition to viremia and viral shedding analyses, this study followed the local and systemic humoral immune response of pigs for 63 days upon inoculation with one of three types of Type-2 PRRSV (PRRSV-2) strains – one modified live virus (MLV) vaccine strain, and two lineage 1 PRRSV-2 strains, NC134 and NC174. The local response was analyzed by quantifying immunoglobulin (Ig)A in nasal swabs. The systemic response was studied by the quantification of IgG with ELISA and homo- and heterologous neutralizing antibodies (NAs) utilizing a novel method of flow cytometry. In all PRRSV-2 inoculated groups, viral nasal shedding started at 3 dpi, peaked between 3 and 7 days post inoculation, and was cleared at 28–35 dpi with sporadic rebounds thereafter. The local IgA response started 4–7 days after viral shedding occurred and showed a bi-phasic course with peaks at 14 dpi and at 28–35 dpi. Of note, the NC134 and NC174 strains induced a much stronger local IgA response. As reported earlier, main viremia lasted from 7 dpi to 28 dpi (NC174), 42 dpi (NC134) or until the end of the study (MLV). Similar to the local IgA response, the systemic IgG response started 4–7 days after viremia; but in contrast to viremia, serum IgG levels stayed high for all PRRSV-2 inoculated groups until the end of the study. A significant finding was that while the serum NA response in the MLV group was delayed by 28 days, serum NAs in pigs infected with our two NC134 and NC174 strains could be detected as early as 7 dpi (NC134) and 14 dpi (NC174). Compared to homologous NA responses, the NA responses against heterologous strains was strong but slightly delayed between our lineage 1 one strains or non-existent between the MLV and lineage 1 strains. This study improves our understanding of the relationship between local and systemic infections and the humoral immune response induced by PRRSV-2 infection or MLV vaccination. Our data also provide novel insights into the timeline of the development of homologous and heterologous NA levels – by both MLV vaccination or infection with two strains from the currently prevalent PRRSV-2 lineage 1.

## Introduction

Despite two decades of commercial vaccination administration to sows and nursery age pigs, Porcine Reproductive and Respiratory Syndrome Virus (PRRSV) continues to distress the global pork industry causing significant losses globally (1, 2) through reproductive failure (3, 4), nursery age pig respiratory distress (5, 6), reduced growth (7), secondary infections (8, 9), and increased mortality (10, 11), as well as, through expenditures on vaccination and biosecurity efforts.

PRRSV is divided into two distinct species: Type 1 (PRRSV-1) and Type 2 (PRRSV-2) (12); furthermore, due to its RNA-viral nature and propensity for genetic mutation, each type is also very diverse so that numerous PRRSV strains can be described as “quasispecies” (13). Based upon the diversity of these quasispecies, the practical vaccination question persists: Will a vaccine induce immunity against a circulating PRRSV strain – so heterologous immunity? And a follow-up question could/ should be: How can we estimate or even determine heterologous immunity? Are phylogenetic analyses sufficient or do we need immunological studies?

Long-term immunity against diseases such as PRRSV is mainly accomplished by two measures – the establishment of cell-mediated immunity via T cells and a protective humoral immune response. We previously characterized the complex homo- and heterologous T-cell response to a lineage 5 modified live virus (MLV) vaccine strain, and two lineage 1 PRRSV-2 strains, a low-pathogenic (LP) NC134 and high-pathogenic (HP) NC174 over the course of 63 days (14).

The complexity of this T-cell response seems to match the humoral immune response: The perplexing relationship between serum NAs, viremia, and protection from PRRSV is highlighted by the numerous reviews of this subject in recent years (15–17). Collectively, these reviews generally agree on two themes: First, serum NA should be investigated for their role in clearance of viremia; and second, vaccination- or infection-generated NAs show limited cross-reactivity to the diversity of PRRSV strains. Three seminal papers further illustrate these points: two serum passive antibody transfer experiments resulted in protection of gilts from reproductive failure (18) and reduction of viremia in young pigs (19); however, following vaccination, NA cross-reactivity between strains is limited to the vaccination strain (20). Additionally, some of these reviews also discuss a delay between the appearance of serum anti-PRRSV IgG (~7–10 dpi) and serum NA (>28 dpi) (15–17). This delay and the overall low vaccination-generated NA titers are a key argument for why current PRRSV vaccinations do not provide heterologous sterilizing immunity. Strain diversity is important because vaccination studies often report undetectable or late developing (>8 weeks post vaccination) NA following vaccination (21–26) or after infection (27–29); this is especially a characteristic of vr-2332 (lineage 5), but not of a lineage 8 vaccine (7, 30). Upon challenge, serum NAs to the vaccination strain are often produced rapidly within 14 dpi (23, 24). PRRSV strain diversity and failure to obtain cross-reactive serum NA titers are generally the reasons postulated for why available PRRSV vaccines improve pig performance but do not provide complete protection or sterilizing immunity to vaccinated pigs (31). Hence, one focus of this study was the cross-reactivity of the humoral immune response induced by MLV vaccination or infection with two PRRSV-2 lineage 1 strains.

In summary, the main purpose of this study was to describe the local and systemic humoral immune response in nursery age pigs to the previously used PRRSV-2 strains (MLV, NC134, and NC174) by quantifying local immunoglobulin (Ig)A in nasal swabs, and systemic IgG and homo- and heterologous neutralizing antibodies (NAs) in serum. We correlated these humoral immune responses to viral shedding and viremia to better assess the humoral immune response before infection and during the active and persistent phases of PRRSV-2 infection. In addition, due to the high variability of PRRSV-2 and the necessity to provide immunity against heterologous strains, we analyzed the NA response not only against the homologous, but also against the other two heterologous challenge strains within a lineage (NC134 vs. NC174) and between lineages (NC134/NC174 vs. MLV). To evaluate the heterology of our strains, we included phylogenetic variation and clustering analyses to determine if they could be used to explain any observed differences in the heterologous immune responses.

Timewise, we will show three things: (i) the local IgA response closely follows viral shedding; (ii) the systemic IgG response also starts shortly after viremia but stays high until the end of the study (63 dpi); and (iii) while the MLV-induced NA response is delayed until 28 dpi as found in the literature, the NC134 and NC174 strains used in this study generated serum NAs within 7 and 14 dpi, respectively, and were cross-reactive against each other.

## Materials and Methods

### Study Design, PRRSV−2 Propagation, Titration, and Sample Processing

The animal trial study design, PRRSV-2 strain source, propagation, titration, and sampling procedures were described previously (14). Briefly, twenty-four 4-week-old weaned pigs from a PRRSV-negative herd (NC State University Swine Education Unit) were moved to the BSL-2 Laboratory Animal Research (LAR) facility at NC State University – College of Veterinary Medicine. Six pigs were randomly assigned into each inoculation treatment group: media (MOCK), intramuscular inoculation with an MLV based on the lineage 5 VR2332 strain (MLV), and intranasal inoculation with either the lineage 1 strains NC134, considered low pathogenic (LP), or NC174, considered high pathogenic (HP). While the use of different routes of administration introduces an additional variable, it best mimics the natural exposure for the NC134 and NC174 field strains and the MLV vaccine strain. Serum samples and nasal swabs were collected prior to inoculation (0 days post inoculation, dpi) and then at 3, 7, 10, 14, 21, 28, 35, 42, 49, 56, and 63 dpi as illustrated in Supplementary Figure 1. Not described previously, for nasal swabs, pigs were restrained and one sterile Puritan Hydraflock swab (Puritan Medical Products Company, Guilford, ME) was inserted into, rotated, and removed from each nostril. The swab end was then placed into a 1.5 ml microcentrifuge tube (Sarstedt, Nümbrecht, Germany) with one ml of sterile phosphate buffered saline (PBS) (Corning, Manassas, VA) cut-off and closed. Upon returning to the laboratory, each 1.5-ml tube was vortexed three times and then the swab end was removed and discarded. The 1.5-ml tubes were stored at −80 °C. Experimental procedures were approved by the NC State University Institutional Animal Care and Use Committee (IACUC) ID# 17-166A.

### Viremia and Serum Anti-PRRSV IgG Analyses

Isolated serum was shipped to Iowa State University Veterinary Diagnostic Laboratory (ISU-VDL) (i) for RT-qPCR analysis of viremia as previously reported (14), and (ii) for the PRRSV X3 enzyme-linked immunosorbent assay (ELISA) to determine serum anti-PRRSV IgG levels.

### Local (Respiratory) Anti-PRRSV IgA and Virus Shedding

Nasal swab extracts (described above) were shipped to ISU-VDL (i) for the PRRSV Oral Fluid IgA ELISA to determine anti-PRRSV IgA presence in the nasal passages, and (ii) for RT-qPCR quantification of PRRSV to determine the active viral shedding from the respiratory system. This method was recently validated for oral fluids (32).

### Neutralizing Antibodies Against Homo- and Heterologous PRRSV-2 Strains

Quantification of NAs was performed by flow cytometry adapted to PRRSV from Käser et al. (33). The full procedure is described below and illustrated in Supplementary Figure 2: 25,000 MA-104 cells (ATCC, Manassas, VA) were seeded in 100 μl Minimum Essential Medium Eagle (MEM, 1x) (Corning) supplemented with 10% Fetal Bovine Serum (FBS) (VWR, Radnor, PA) and 1x penicillin/streptomycin (Corning) into a 96-well flat bottom plate (Sarstedt) and incubated for 24 h at 37°C and 5% CO_2_. To determine the NA presence in serum, the serum was heat inactivated and mixed with each of the PRRSV-2 strains [MOI 0.1] at a 1:8 dilution. This MOI was selected because it resulted in ~60–80% infection in our target MA-104 cells for all virus strains. Then, 100 μl of this mixture was added to each well of MA-104 cells and incubated for another 24 h. Each serum sample from each animal at each time point was tested against all three virus strains for the % infection at a 1:8 serum dilution. This dilution was selected after a preliminary analysis showed that a 1:8 dilution provided the best data distribution: It reached the upper limits of the assay only for high-level sera and >21 dpi and provided a high sensitivity even for the low-level MLV NA sera (d.n.s.).

For FCM, after 24 h of MA-104 incubation with the serum / virus mixture, the media was removed, and cells were washed with PBS. MA-104 cells were isolated from the plate using 0.25% trypsin (Corning) and duplicate wells were combined and transferred to a round-bottom 96-well plate. For the FCM staining, cells were stained with Live / Dead near-IR (Invitrogen, Eugene, Oregon) and then fixed and permeabilized with eBioscience Foxp3/Transcription Factor Staining Buffer set (Invitrogen, Carlsbad, CA). After fixation and permeabilization, cells were intracellularly stained for PRRSV infection using anti-PRRSV SR-30A (RTI LLC, Brookings, SD) directly conjugated in house to Alexa Fluor 647 (Invitrogen). Cells were recorded on a Cytoflex using the CytExpert software (Beckman Coulter, Brea, CA). Data analysis was performed with FlowJo version 10.5.3 (Becton Dickinson, BD) with gates based upon FMO controls. The % suppression was calculated by comparing the % infection at a given day post inoculation (x dpi) with the % infection at 0 dpi using the following formula:

$$\begin{array}{l} \%\;Suppression=100-\left(\;\frac{\%\;infection\;\left(x\;dpi\right)}{\%\;infection\;\left(0\;dpi\right)}\right)x\;100 \end{array}$$

Based on the MOCK sera (mean % suppression + 3 x standard deviation), we set the threshold for a positive NA result at 24.55.

### Neutralizing Antibody Verification and Determination of Positive Samples

To further verify results obtained by our in-house NA quantification, we sent selected serum samples to the South Dakota State University Animal Research and Diagnostic Laboratory (SDSU ARDL) to perform a fluorescent focus neutralization (FFN) test – an accepted standard for NA titration. The SDSU ARDL determines a positive titer as the highest serum dilution with a 90% or higher reduction in the number of fluorescent focus forming units (34). A comparison between the results obtained by our FCM and the SDSU ARDL FFN test show a remarkable similarity as shown in Table 1: While each of the tests detected NAs earlier than the other in two instances, both tests detected NAs at the same time point in eight out of twelve pigs.

**Table 1:** Validation of flow cytometry NA method by comparison with SDSU ADRL FFN results.

**Animal**	**NA Method**	**0 dpi**	**7 dpi**	**10 dpi**	**21 dpi**	**49 dpi**
LP 1	FFN	<1:4	1:4	1:512	1:256	1:512
	FCM	0	23.23	70.15	99.37	99.89
LP 2	FFN	<1:4	<1:4	1:4	1:128	1:128
	FCM	0	6.7	31.34	93.73	99.96
LP 3	FFN	<1:4	1:64	1:32	1:512	1:512
	FCM	0	72.77	90.5	98.8	99.96
LP 4	FFN	<1:4	1:256	1:256	1:256	1:256
	FCM	0	84.7	90.03	99.59	99.96
LP 5	FFN	<1:4	1:32	1:512	1:512	1:512
	FCM	0	78.57	96.13	99.52	99.88
LP 6	FFN	<1:4	<1:4	1:4	1:512	1:256
	FCM	0	−6.64	5.9	94.2	99.58
HP 1	FFN	<1:4	<1:4	1:4	1:32	1:512
	FCM	0	0.67	25.74	83.42	99.42
HP 2	FFN	<1:4	<1:4	1:4	1:256	1:256
	FCM	0	−8.01	72.44	99.7	99.96
HP 3	FFN	<1:4	<1:4	<1:4	1:256	1:512
	FCM	0	3.67	10.72	99.6	99.93
HP 4	FFN	<1:4	<1:4	<1:4	1:8	1:512
	FCM	0	75.56	74.96	74.52	99.97
HP 5	FFN	<1:4	<1:4	<1:4	1:256	1:512
	FCM	0	3.54	6.93	99.66	99.91
HP 6	FFN	<1:4	<1:4	<1:4	1:16	1:512
	FCM	0	11.34	27.76	97.61	99.6

*Green shaded fields represent a positive result. FFN depicts the titer value; the FCM values show the % suppression*.

Both assays detected serum NAs as early as 7 dpi; and the days in which NA changed from negative (white fields) to positive (green fields) were the majority the same in both methods. Despite these similarities, our in-house flow cytometry (FCM) assay requires and will soon undergo a full validation procedure.

### Phylogenetic Analyses

We built a comprehensive genetic database comprised by PRRSV-2 ORF5 gene sequences obtained from GenBank along with the location (US state) as metadata. This gene was chosen due to its extended use in literature for phylogenetic studies. Sequences were aligned using Mega X, available at www.megasoftware.net (35). The recombination detection program (RDP) v4 (36) was used to search for evidence of recombination within our dataset using six different methods (BootScan, Chimera, MaxChi, RDP, 3Seq, and SiScan). Recombinant sequences were detected and removed. After recombinant sequences were removed and to avoid location-bias, five sequences from each state were randomly chosen to perform this analysis. In the case of NC, we also added three target sequences that have been used in laboratory experiments over time to compare with the currently circulating field samples. These were the only three sequences where the strain was known (PRRSV-2 NC174, NC134, and VR2332). This selection led the final database with a total of 93 sequences.

The phylogenetic tree was generated by a discrete phylogeography estimation via Bayesian inference through Markov Chain Monte Carlo (MCMC), implemented in BEAST v2.6.0 (37), applying a GTR model with gamma-distributed rate heterogeneity among sites. To determine the best fitting model of evolution we used PhyML with Smart Model Selection (SMS) (38). Since all sequences are from the same species, we assumed a constant mutation rate and applied a strict clock model.

All analyses were developed for 200 million generations, sampling every 10,000th generation and removing 10% as chain burn-in. The Markov Chain Monte Carlo analysis was investigated using Tracer software v1.7 (39) to ensure adequate effective sample sizes (ESS) (above 200). Final trees were summarized and visualized via Tree Annotator v. 2.3.0 and FigTree 1.4.3, respectively (40, 41). Finally, we used SpreaD3 to visualize the transmission routes through the country (42).

To assess potential gathering within the RFLP NC174 and NC134 clades, these were identified and isolated from the original database, but only few states had data available (data not shown).

### Statistical Analysis

Statistical analyses were performed using Graphpad Prism 9 (Graphpad Software, San Diego, CA). The qPCR data for virus quantification were log-transformed prior to statistical analysis. Data throughout the study were analyzed using a repeated-measures two-way ANOVA with a Geisser-Greenhouse correction. Multiple comparisons were performed using a Tukey test.

## Results

The clinical signs and viremia of this study were reported previously (14). While all MOCK-inoculated pigs stayed PRRSV-negative, viremia was detected in all PRRSV-inoculated pigs at the first evaluated time point – 7 dpi. Viremia peaked between 7 and 14 dpi and cleared between 35 dpi (HP), 49 dpi (LP), or a steady low-level viremia (MLV). After clearance, some animals showed sporadic rebounds of viremia. In contrast to the healthy pigs in the MOCK and MLV groups, most LP and HP infected pigs exhibited clinical signs consistent with PRRSV-2 infection – fever, lethargy, and sneezing (14).

### Local PRRSV Replication and Nasal Shedding of PRRSV

**Figure 1:**
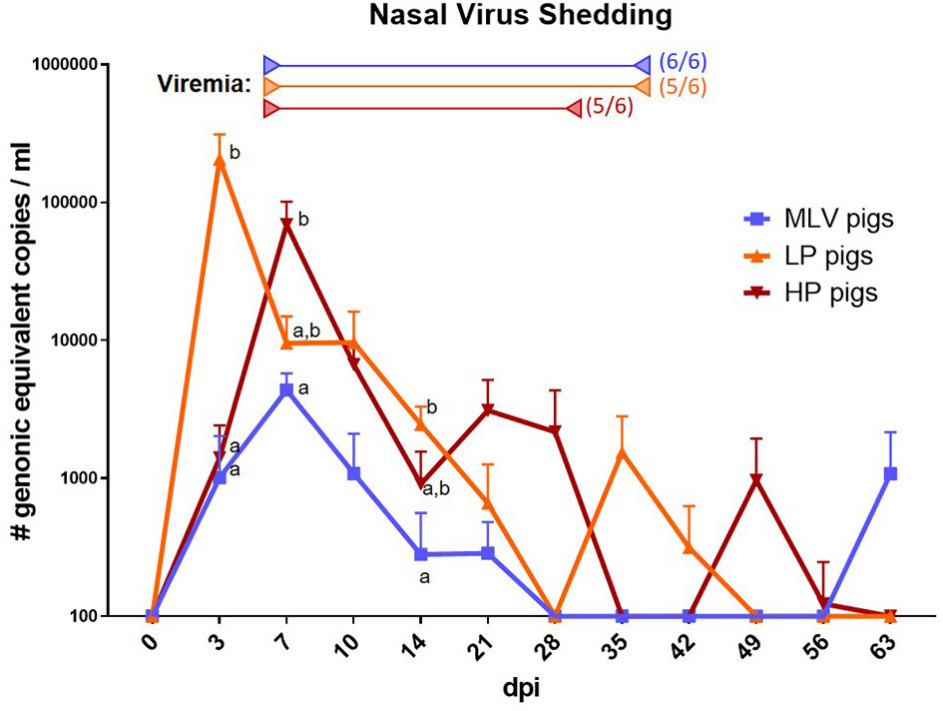
Nasal shedding of PRRSV. To assess the shedding of PRRSV, virus prevalence was quantified in nasal swabs via RT-qPCR in MLV pigs (blue line), LP pigs (NC134, orange line) or HP pigs (NC174, red line). Start of viremia for each group is indicated by the left arrows in the respective colors. All pigs became viremic during the study. The right arrows show the last dates at which most animals were still viremic; the numbers besides these arrows indicate the number of positive animals on that day. The graph illustrates the mean ± standard error of the mean (SEM). MOCK pigs were PRRSV-negative at each time point. 100 (y-axis) is representative of a negative value. The virus qPCR data was log-transformed for readability. A repeated-measures two-way ANOVA with a Geisser-Greenhouse correction and Tukey test were utilized for differences between groups at each time point; groups with dissimilar superscripts (a or b) are significantly different (*p* < 0.05).

Local PRRSV replication and the resulting nasal shedding was analyzed by PRRSV RT-qPCR quantification in nasal swabs. Pooled samples from MOCK pigs were PRRSV negative throughout the study (data not shown, d.n.s.). For MLV and LP pigs, nasal shedding generally preceded viremia (Figure 1): While viremia peaked at 14 dpi, nasal shedding started at three dpi with peak shedding at 3 dpi (LP) or 7 dpi (MLV). In these MLV and LP pigs, the drop in viral shedding also preceded the clearance of viremia: While viremia could be detected until 42 dpi for LP pigs and until the end of the study for MLV pigs, PRRSV loads in nasal swabs from MLV and LP pigs dropped below the detection level by 28 dpi. For HP pigs, nasal shedding of PRRSV generally corresponded with viremia: Both peaked at 7 dpi and were cleared by 35 dpi with some sporadic post-clearance rebounds. Some pigs within all groups showed sporadic nasal shedding of PRRSV even after initial viral clearance. These data demonstrate that PRRSV-infected pigs quickly shed the virus; and they confirm that even after initial clearance, pigs can shed PRRSV for an extended period of time.

### Local Anti-PRRSV Humoral Response

**Figure 2:**
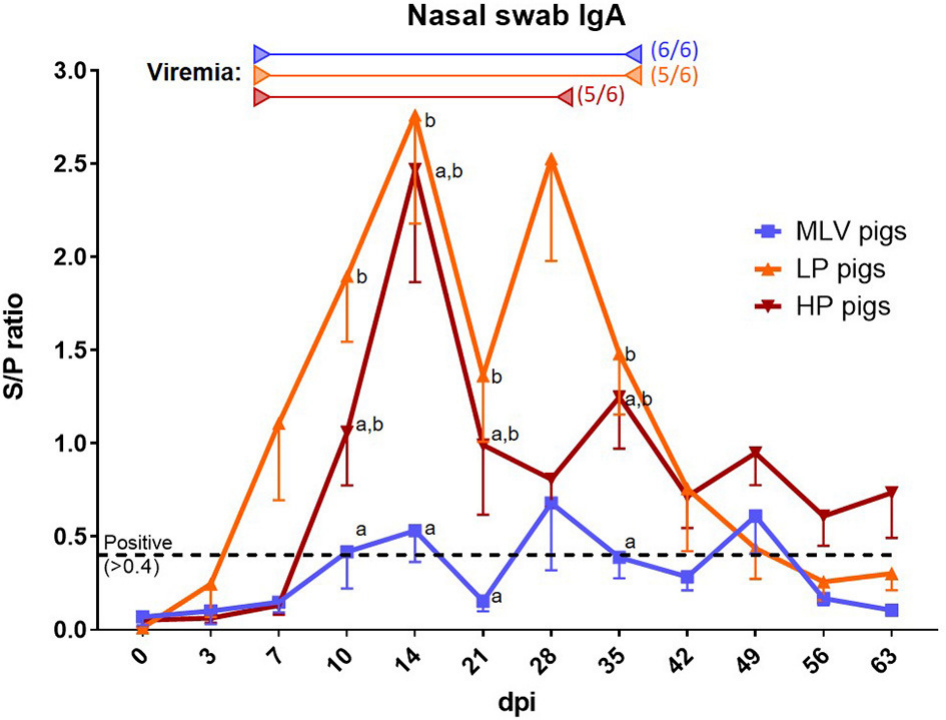
The local anti-PRRSV-2 antibody response. To assess the local anti-PRRSV antibody response, anti-PRRSV IgA antibodies were quantified in nasal swabs via ELISA in MLV pigs (blue line), LP pigs (NC134, orange line) or HP pigs (NC174, red line). Start of viremia for each group is indicated by the left arrows in the respective colors. All pigs became viremic during the study. The right arrows show the last dates at which most animals were still viremic; the numbers besides these arrows indicate the number of positive animals on that day. The graph illustrates the mean ± SEM. The cut-off for positivity is indicated by the black dashed lined at an S/P ratio of 0.4 and was set for oral fluids, not nasal swabs. Based on the potentially higher dilution of the nasal swabs, the S/P values might be underestimated. MOCK pigs were negative at each time point (not shown). A repeated-measures two-way ANOVA with a Geisser-Greenhouse correction and Tukey test were utilized for differences between groups at each time point; groups with dissimilar superscripts (a or b) are significantly different (*p* < 0.05).

As for the viremia analysis, nasal swabs were used to determine the local anti-PRRSV humoral immune response via anti-PRRSV IgA quantification by ELISA (S/P ratio, Figure 2). Of note, this assay was developed for oral fluids and based on a potential higher dilution in these nasal swabs, their S/*P*-values might be underestimated. Pigs within the MLV groups barely crossed the threshold for positive S/P ratios of 0.4 with maximum ratios of ~0.7 (blue line). In contrast, nasal swabs from LP and HP inoculated pigs had with ~2.5 very high levels of IgA. Both groups also showed an interesting bi-phasic curve: The first peak appears at 14 dpi and the second peak at 28 dpi for LP pigs and 35 dpi for HP pigs. While the HP nasal IgA levels stayed positive until the end of the study, they became negative for MLV and LP pigs at 56 dpi.

### Systemic Anti-PRRSV Humoral Response

**Figure 3:**
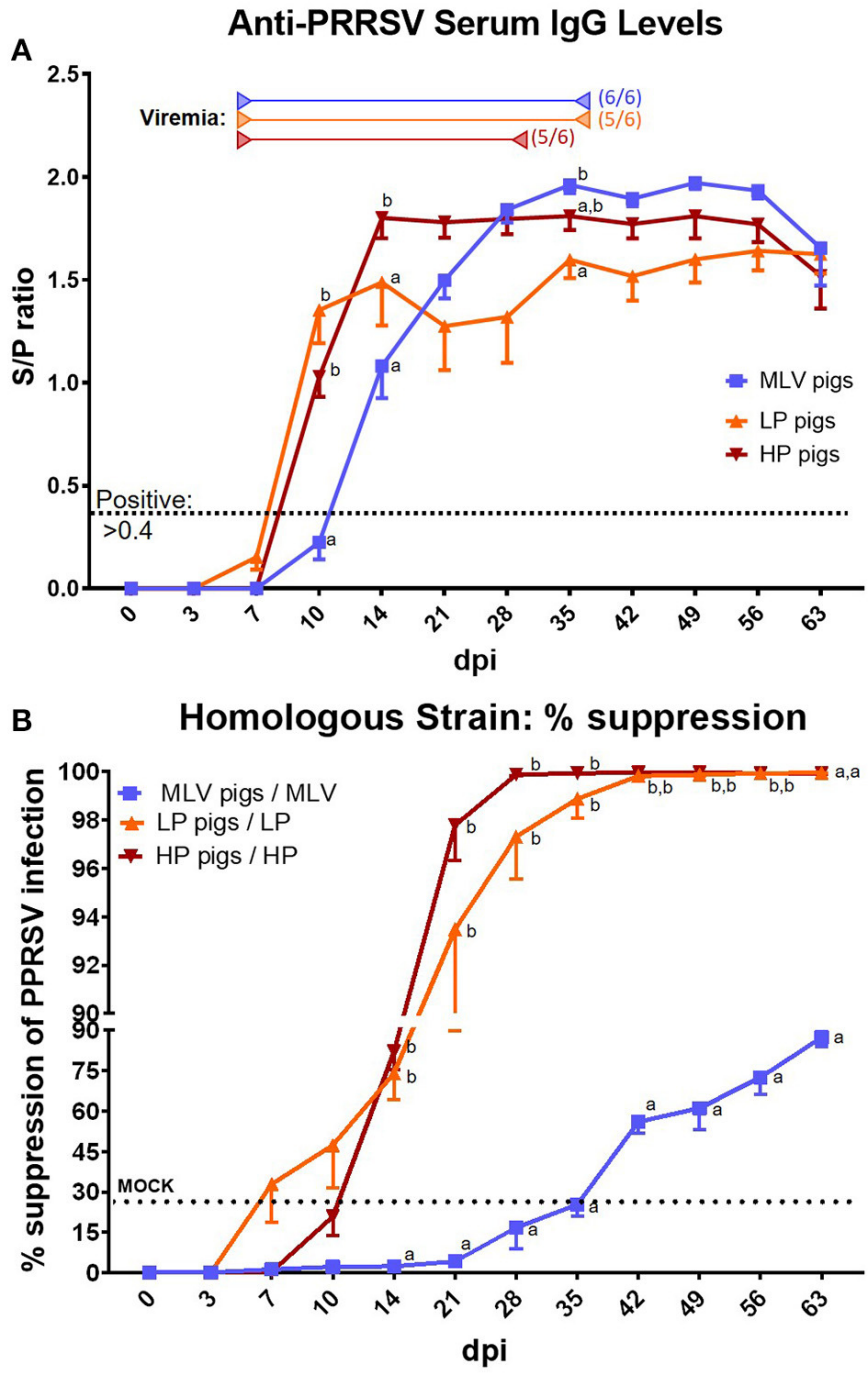
The systemic anti-PRRSV-2 humoral immune response – IgG titers and homologous neutralizing antibody levels. **(A)** Serum IgG levels determined by ISU VDL with ELISA X3. MOCK pigs were PRRSV-negative at each time point (not shown). The cut-off for positivity is indicated by the black dotted lined at an S/P ratio of 0.4. MOCK pigs were negative at each time point (not shown). Start of viremia for each group is indicated by the left arrows in the respective colors. All pigs became viremic during the study. The right arrows show the last dates at which most animals were still viremic; the numbers besides these arrows indicate the number of positive animals on that day. **(B)** % Suppression of PRRSV infection by serum NAs in a 1:8 dilution against the homologous inoculation strain (MOI 0.1). The graph illustrates the mean ± SEM. The dotted “MOCK” line at 24.55% suppression represents the cut-off for a positive value based on MOCK animals (mean % suppression + 3x standard deviation). A repeated-measures two-way ANOVA with a Geisser-Greenhouse correction and Tukey test were utilized for differences between groups at each time point; groups with dissimilar superscripts (a, b or c) are significantly different (*p* < 0.05).

**Figure 4:**
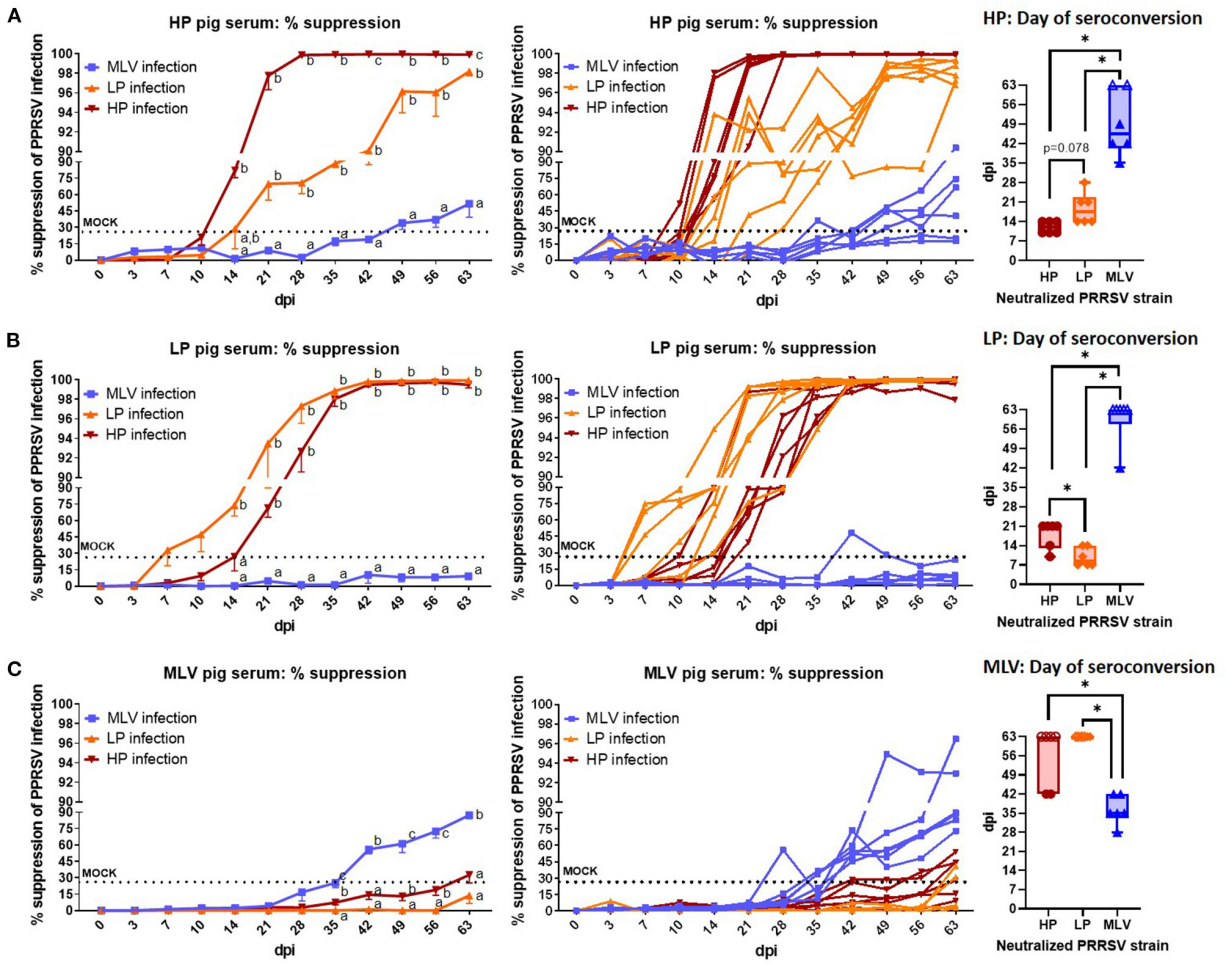
The systemic neutralizing antibody response to heterologous PRRSV-2 strains. Percent suppression of PRRSV infection by serum NAs in a 1:8 dilution against the homologous and heterologous PRRSV strains (MOI 0.1). The neutralizing capacity of serum from HP-inoculated pigs **(A)**, LP-inoculated pigs **(B)**, or MLV-inoculated pigs **(C)** was tested against MLV virus (blue lines/ box plots), LP virus (orange lines/ box plots), and HP virus (red lines/ box plots). Left graphs show the mean ± SEM, the middle graphs show the single values of the pig serum NA capacity to suppress % infection of the homologous and two heterologous strains; so each line represents the neutralization capacity of serum from one animal. The dotted “MOCK” line at 24.55% suppression represents the cut-off for a positive value based on MOCK animals (mean % suppression + 3x standard deviation). The Min-to-Max Box plots (+all data points and the Mean) illustrate the days on which the pigs NA levels seroconverted – became positive according to the 24.55% suppression cut-off. Open symbols at 63 dpi represent animals that did not seroconvert against the respective PRRSV strain. A repeated-measures two-way ANOVA with a Geisser-Greenhouse correction and Tukey test were utilized for differences between groups at each time point. Groups with dissimilar superscripts (a, b or c) are significantly different (*p* < 0.05). For the box plots, *indicates significant differences (*p* < 0.05).

The systemic humoral immune response to PRRSV was evaluated in two ways – serum anti-PRRSV IgG levels (Figure 3A) and the quantification of homologous (Figure 3B) and heterologous (Figure 4) NAs.

### Serum IgG Levels

Serum IgG levels are depicted in Figure 3A. Within MLV pigs, serum anti-PRRSV IgG levels became positive by 14 dpi and they reached their highest levels ~28–35 dpi – S/P ~1.9–2.0. Both, LP and HP pigs reached positive serum anti-PRRSV IgG levels slightly earlier at 10 dpi: LP pigs peaked at 14 dpi at an S/P ratio of ~1.5 and HP pigs peaked at 14 dpi with an S/P ~1.8. Only toward the end of the study, both the serum IgG levels in MLV and HP pigs dropped to an S/P ratio of 1.5 – the same level as LP pigs. So, in conclusion, LP and HP pigs had a faster and significantly stronger serum IgG response at the earlier time points (10 & 14 dpi); but at later time points, either MLV or HP pigs had a stronger response than LP pigs before they all arrive at the same level at the end of the study (63 dpi).

### Neutralizing Antibodies Against the Homologous PRRSV Strain

Neutralizing antibodies against the homologous PRRSV strain are depicted in Figure 3B. While positive homologous serum NA levels could not be detected in MLV pigs until 42 dpi, both LP and HP pigs showed a fast induction of homologous serum NAs by 7 or 14 dpi, respectively. At the chosen 1:8 serum dilution, MLV pigs never reached 90% neutralization rates; in contrast, HP and LP pigs even surpassed 99% of neutralization by 28 or 42 dpi, respectively. As mentioned before, these data including the induction of serum NAs as early as seven dpi were corroborated by SDSU ARDL using an FFN test – the gold standard for PRRSV NA quantification (Table 1).

### Neutralizing Antibodies Against the Heterologous PRRSV Strains

Neutralizing antibodies against the heterologous PRRSV strains are depicted in Figure 4. With respect to the effect of NA against heterologous PRRSV-2 strains, HP and LP pigs exhibit strong cross-reactivity between strains as illustrated in Figure 4B. While serum from pigs infected with HP virus neutralized LP virus as early as 21 dpi, it took until the end of the study to reach their maximum neutralization of ~98% (Figure 4A, left graph); also, while most HP pigs reached the plateau phase against the homologous HP strain by 28 dpi, it took them until 49 dpi to neutralize >96% of the LP virus (Figure 4A, middle graph). On average, HP pigs seroconverted for homologous NAs at 12 dpi which is at least by number shorter compared to the mean heterologous NA conversion against the LP strain which occurred at 18.7 (*p* = 0.078, Figure 4A, right graph). For serum from LP pigs, this cross-reactivity to HP virus had a statistically significant delay with an average day of seroconversions against the homologous LP strain at 9.8 days and against the HP stain at 18 days (Figure 4B, right graph). In summary, the within-lineage 1 heterologous neutralization lagged ~1 week behind the homologous response (Figure 4A, B, right graphs). Regarding cross-reactivity of serum NAs from LP and HP infected pigs against the MLV virus strain, HP sera showed only low-level of NAs against MLV and only after 49 dpi (Figure 4A); and with the exception of one pig at one time point (Figure 4B, right side), LP pigs never developed a positive NA response against MLV virus (Figure 4B). Vice versa, the heterologous serum NA response from MLV pigs also showed only a barely detectable neutralization capacity against both LP and HP viruses and only in some animals (Figure 4C). These data illustrate that the HP and LP strains induced a robust, progressing heterologous NA response within the PRRSV-2 lineage 1; but across lineages, this heterologous NA response was minimal to non-existent.

### Phylogenetic Analyses

**Figure 5:**
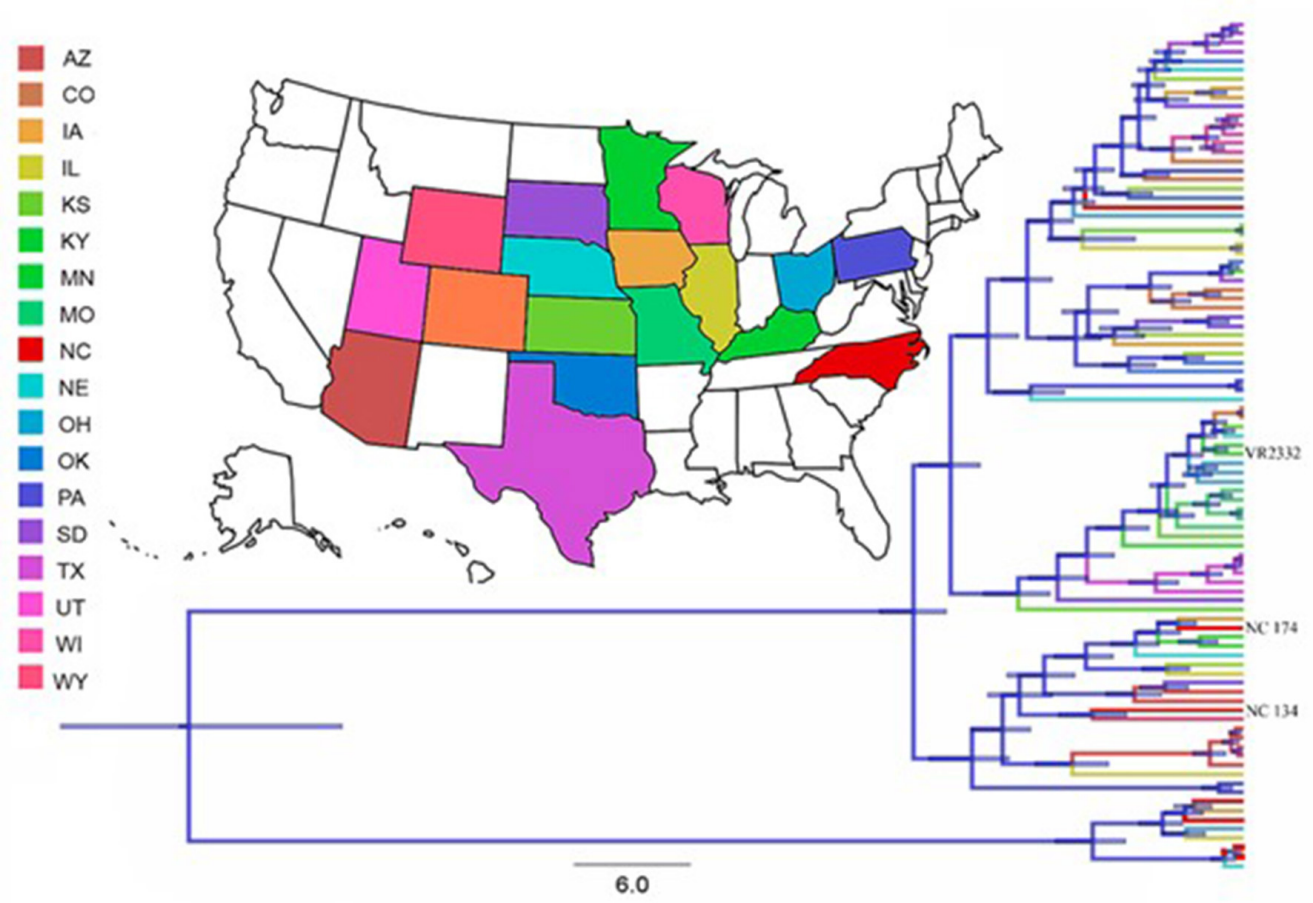
Phylogenetic tree of the 93 PRRSV-2 sequences of the studied database. Tree branches are colored based on the reported state where the sequence was collected. Node bars represent posterior probabilities of branching events (*p* > 0.95). These analyses showed that the most likely origin of the analyzed sequences is in Pennsylvania, with a Root State Posterior Probability (RSPP) of 0.28 followed by North Carolina (RSPP = 0.21).

The generally accepted timeline for the NA response to PRRSV is delayed – starting at ~28 dpi (16, 17). As evidenced in Figure 3, the homologous NA response to the two lineage 1 strains used in this study occurred within 14 dpi; and the heterologous cross-reactivity to the other lineage 1 strain was much higher than to the lineage 5 strain. To explain these different humoral immune responses and to better integrate them with data reported in the literature, we performed phylogenetic analyses from 93 PRRSV-2 ORF5 sequences. Thereby, we planned to test our hypothesis that our NC134 and NC174 strains will form a very tight and new cluster. Despite the different lineages, the ORF5 similarity between strains showed a less dramatic difference between the strains (ISU VDL sequenced and/or BLAST analysis): NC174 vs. NC134 (86%); NC174 vs. VR2332 (87.9%); and NC134 vs. VR2332 (84.8%). As shown in Figure 5, the obtained phylogeny is divided in two main clusters: The smallest one is formed by eight sequences with four coming from North Carolina, and one each from Iowa, Illinois, Ohio, and Nebraska; the second cluster is formed by the remaining 85 sequences. So, the results obtained in the phylogenetic study did not reflect the NA data from this study.

## Discussion

The effect of the humoral immune response on lung pathogens like PRRSV is complex. Local antibodies in the respiratory tract can prevent initial infection; and they limit shedding and lung disease. Due to their active transport through the lung epithelium, IgAs are a major contributor to this local antibody response. In contrast, IgG contributes mostly to serum antibody levels. Thereby, IgGs mainly limit viremia and the systemic spread of PRRSV. And within both IgA and IgG, NAs play a crucial part of the antibody response due to their ability to block viral infection. In addition to these complex roles and interactions, the immunosuppressive capacities and high mutation rates of PRRSV further complicate the understanding of this humoral immune response: The high mutation rates of PRRSV allow emerging virus strains to evade the humoral immune response – an effect often seen with commercial MLV vaccines (21–26); and immunosuppression seems to limit and delay the development of NAs (16, 17). The goal of this study was to investigate these complex interactions and the role of the local and systemic immune responses induced by intramuscular vaccination using a lineage 5 based MLV PRRSV-2 strain and intranasal inoculation with two lineage 1 PRRSV-2 strains of different virulence – NC134 and NC174. As mentioned in the introduction, these strains were selected to facilitate the study of the heterologous NA responses within currently prevalent PRRSV-2 lineage 1 strains and across lineages between the lineage 5 MLV strain and the two lineage 1 strains.

We have previously shown that inoculation with each of the three strains induces viremia within 1 week (14). This is in line with the current understanding of the development of PRRSV viremia (16). In this study, we observed that nasal viral shedding also occurs very quickly after inoculation – within 3 dpi (Figure 1). Shedding peaked within the first week of infection and lasted for 3–5 weeks with sporadic shedding throughout the rest of the study – until 9 weeks post infection (Figure 1). Eclercy et al. recently reported that PRRSV-1 infection sheds nasally in a similar manner (43). Combined, these data indicate that both PRRSV-1 and PRRSV-2 show a similar timeline of lung propagation and nasal viral shedding. Compared to the lineage 1 PRRSV-2 strains used in this study, the MLV strain had a significantly lower peak viral load in nasal swabs – only about 1/10th. This indicates that compared to the NC134 and NC174 strains, MLV replicates considerably less in porcine lung tissue. This is most likely caused by one of three mechanisms – attenuation, strain diversity or by the different route of administration (intranasal for NC134 and NC174 vs. intramuscular for MLV). Conclusively, these data indicate that each of those PRRSV-2 strains quickly replicates in lung tissue and leads to nasal shedding within 1 week; this shedding lasts for several weeks and can flare up even after the active phase of viremia; and while the MLV strain leads to lower lung replication and shedding, MLV shedding can also occur even 9 weeks post infection.

Shortly after the start of viral shedding, the local IgA response started in all groups – within 1–2 weeks (Figure 2). Interestingly, this local IgA response had a bi-phasic curve peaking at 2 and 4–5 weeks post infection. While this method should be considered rather semi-quantitative, this curve supports future studies into the potential relevance of and biology behind this pattern. Furthermore, the biphasic IgA curve mimics and trails the biphasic viremia observed mainly for the NC174 strain in the blood (14). Like nasal shedding, the local IgA response also had sporadic flare-ups after initial clearance of the local infection. As well, the lower lung replication of the MLV strain is also reflected in a lower induction of local IgA – Figure 2: Peak S/P ratio of ~0.7 for MLV vs. ~2.5 for NC134 and NC174. Again, the lower lung response can be explained not only by strain diversity but also by MLV attenuation with a lower replication in macrophages and the different routes of administration. These data contribute to our limited understanding of the local humoral immune response to PRRSV-2; and they provide a detailed timeline on the local IgA response to a vaccine and two prevalent PRRSV-2 strains.

The systemic IgG response to PRRSV-2 has been extensively studied and reviewed (16, 17). Our data align with previous reports showing an induction of serum IgG levels developing between 1 and 2 weeks post infection (Figure 3A). In contrast, the majority of studies report a delayed systemic NA response after at least 28 dpi (16, 17). Pigs vaccinated with the MLV strain based on the lineage 5 reference strain VR2332 showed a similarly delayed homologous NA response that was first detected at 42 dpi (Figure 3B, blue line). Yet, in contrast, our PRRSV-2 lineage 1 strains NC134 and NC174 developed serum NAs within 1–2 weeks (Figure 3B, orange and red lines, and Table 1). Two recent studies reported NAs prior to 28 dpi: (i) The Faldyna group reported in both vaccinated and non-vaccinated pigs high NA titers (~70) at 21 days post challenge – their earliest reported post-challenge date (44); and (ii) the Mateu group also reported serum NAs in 4/4 animals infected with the 3,267 PRRSV-1 strain within 21 dpi – also their earliest reported time point (45). The high titers in the Faldyna study indicate that they might have also detected low-level NAs at an earlier time point.

The frequently cited study by Yoon et al. from 1994 also shows the induction of low-level NAs before 28 dpi: (i) Upon addition of fresh pig serum, NAs appeared as early as 9 dpi; and (ii) at 14 dpi (the earliest time point reported in their Table 3) they detected a low NA titer of 4 (27). The addition of fresh pig serum in (i) indicates a role of complement in the detected “neutralization.” In our study, it is highly unlikely that complement played a role in virus neutralization since both our own flow cytometry protocol as well as the well-established FFN protocol at SDSU includes a complement inactivation step prior to adding the serum to the virus. Yoon et al. could eliminate the neutralization activity of the NAs detected at 14 dpi by adding anti-swine IgM antibodies prior to the NA assay; this indicates that these early NAs likely belong to the IgM isotype (27). This assumption seems reasonable since we previously showed that the T-cell response including the CD4 T-cell response that is a main contributor to the isotype class switch on B cells takes ~2 weeks to establish (14). Conclusively, while the development of NAs against specific PRRSV strains has been reported before, we have identified two PRRSV-2 lineage 1 strains with the rare ability to induce NAs within 1–2 weeks. With an ~1-week delayed onset, these NAs have also shown to be cross-reactive with at least one member of the PRRSV-2 lineage 1.

In contrast to the within-lineage heterologous reactivity, cross-lineage neutralization was very weak: the LP strain did not develop NAs against the MLV strain (Figure 4B, orange vs. blue line); and only two animals in the MLV group developed minimal NAs against the LP strain (Figure 4C, blue vs orange line). Infection with HP led to the development of some low-level NAs against the MLV strain after 49 days – a very long 35-day delay considering the homologous NAs in HP pigs reached higher levels already at 14 dpi. The lack of NAs or the very late NA development (>8 weeks post vaccination) has repeatedly been reported post vaccination (21–26) or post infection (27–29). In addition, PRRSV strain diversity and failure to obtain cross-reactive serum NA titers are generally the reasons postulated for why available PRRSV vaccines improve pig performance but do not provide complete protection or sterilizing immunity to vaccinated pigs (31).

Therefore, these NA data indicate that while heterologous protection through NAs may be achievable within NC134 and NC174 strains, cross-lineage protection by the MLV strain is less likely conferred through NAs but rather through the induction of a more cross-reactive T-cell response (14).

In the introduction we asked the question if phylogenetic studies are sufficient to estimate/ determine heterologous immunity. We tried to answer this question for the PRRSV strains included in this study; based on a phylogenic analysis of PRRSV strains in the US (46), we hypothesized that our North Carolina lineage 1 strains will form a specific cluster that could explain the different timeline in the induction of neutralizing antibodies. Yet, our own phylogenetic analysis shows that while these two NC strains are more closely related to each other than to the VR2332-based MLV strain, they shared a cluster with ten other PRRSV-2 strains (Figure 5). While we had to reject our hypothesis based on this analysis, it demonstrates that a phylogenetic analysis alone is inadequate to determine the immunogenicity and cross-reactivity of the immune response of different PRRSV strains. Furthermore, based on the limited cross-lineage reaction of NAs in this study and the successful cross-lineage T-cell response between those exact strains (14) we can conclude that a comprehensive analysis of the immune response to PRRSV should include both, the humoral and the cellular immune response.

## Summary/Conclusion

**Figure 6:**
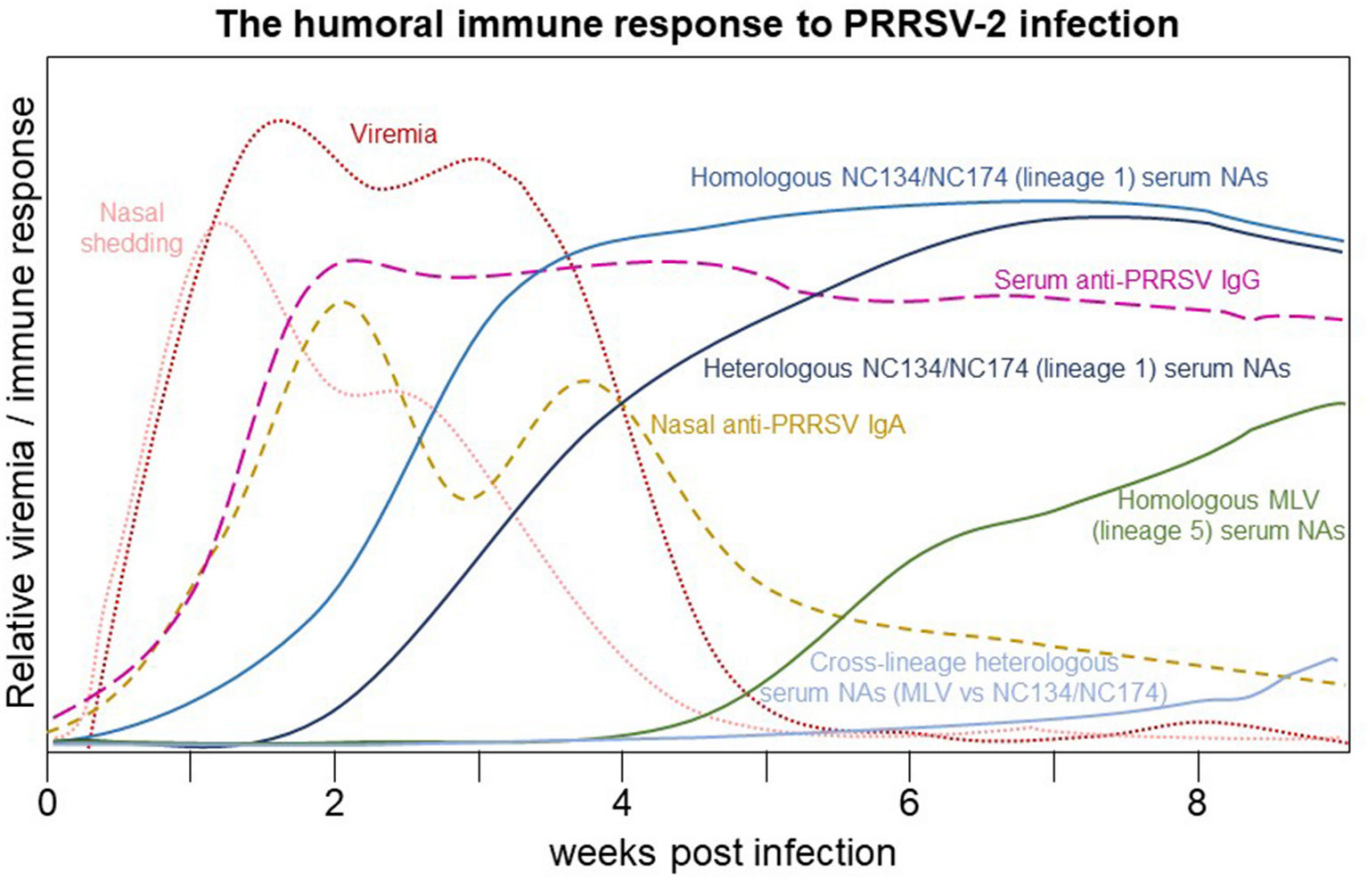
The humoral immune response to PRRSV-2 infection. After infection, viremia and shedding increase to a maximum within 1–2 weeks post infection (wpi). By 2 wpi, anti-PRRSV serum IgG and nasal IgA reach a maximum: nasal IgA slowly declines during viremia and then gradually decreases by 9 wpi while serum IgG remains near the maximum value through 9 wpi. Homologous serum NAs increase to a potentially sterilizing level around 3 wpi while heterologous lineage 1 serum NA achieve the same level between 4 and 6 wpi. Homologous serum NAs to the vaccination lineage 5 strain approach a probably sterilizing range at or after 9 wpi; however, their cross-reactivity with lineage 1 strains remains minimal through 9 wpi.

In addition to providing a timeline for viral shedding by PRRSV-2 strains, this study provides an overview of the humoral immune response to PRRSV-2 infections with a VR-2332-based lineage 5 strain and two lineage 1 strains – NC134 and NC174 of different virulence. This humoral immune response is summarized in Figure 6. We show that both the local IgA and systemic response closely follow viral shedding; but in contrast to the local IgA response, the systemic IgG response stays high until the end of the study (63 dpi). We furthermore described two lineage 1 PRRSV-2 strains with the rare ability to induce serum NAs within 1–2 weeks post infection; these antibodies showed only a shortly delayed within-lineage cross-reactivity. The early induction of NAs with cross-reactivity against at least one strain of the very prevalent PRRSV-2 lineage 1 indicates a potential use of these strains to develop novel PRRSV-2 vaccines.

## Data Availability Statement

The raw data supporting the conclusions of this article will be made available by the authors, without undue reservation.

## Ethics Statement

The animal study was reviewed and approved by NC State University Institutional Animal Care and Use Committee (IACUC) ID# 17-166A.

## Author Contributions

JF, GA, and TK: conceptualization. AK and TK: data curation, formal analysis, methodology, and visualization. JF, GA, and TK: funding acquisition. AK, AA, AF, LC, EC, GA, and TK: investigation. GA and TK: project administration. AK, LC, GA, and TK: resources. EC, GA, and TK: supervision. AK, AA, AF, LC, JF, EC, GA, and TK: validation and writing—review & editing. AK: writing—original draft. All authors contributed to the article and approved the submitted version.

## Conflict of Interest

The authors declare that the research was conducted in the absence of any commercial or financial relationships that could be construed as a potential conflict of interest.
