# Supplementary material for: The Local and Systemic Humoral Immune Response Against Homologous and Heterologous Strains of the Type 2 Porcine Reproductive and Respiratory Syndrome Virus
Source: Front Immunol. 2021 Mar 9;12:637613. doi: 10.3389/fimmu.2021.637613 (PMC7985350; doi:10.3389/fimmu.2021.637613)
Supplement: Supplementary file 1 [file Image_1.pdf]

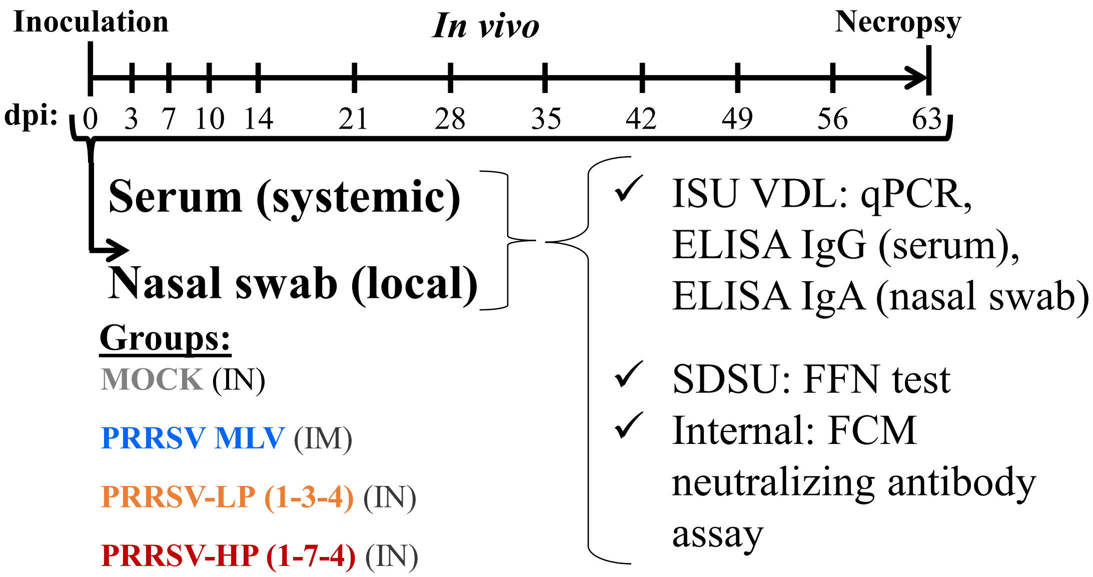

Supplementary Figure 1. Study design. At 0 days post infection (dpi), six pigs per group were inoculated intranasally with PBS (MOCK), a low-pathogenic (LP) NC134 or a high-pathogenic (HP) NC174 PRRSV-2 strain. PRRSV MLV pigs were inoculated intramuscularly with a VR2332-based modified live virus strain. On the indicated days, serum and nasal swabs were taken to analyze the systemic and local viral loads and antibody response. PRRSV-specific RT-qPCR, IgG and IgA were analyzed by the Iowa State University Veterinary Diagnostic Laboratory (ISU VDL). The FFN tests were performed by South Dakota State University. The flow cytometry (FCM) quantification of Nas was performed in house.
