# Supplementary material for: The Local and Systemic Humoral Immune Response Against Homologous and Heterologous Strains of the Type 2 Porcine Reproductive and Respiratory Syndrome Virus
Source: Front Immunol. 2021 Mar 9;12:637613. doi: 10.3389/fimmu.2021.637613 (PMC7985350; doi:10.3389/fimmu.2021.637613)
Supplement: Supplementary file 2 [file Image_2.pdf]

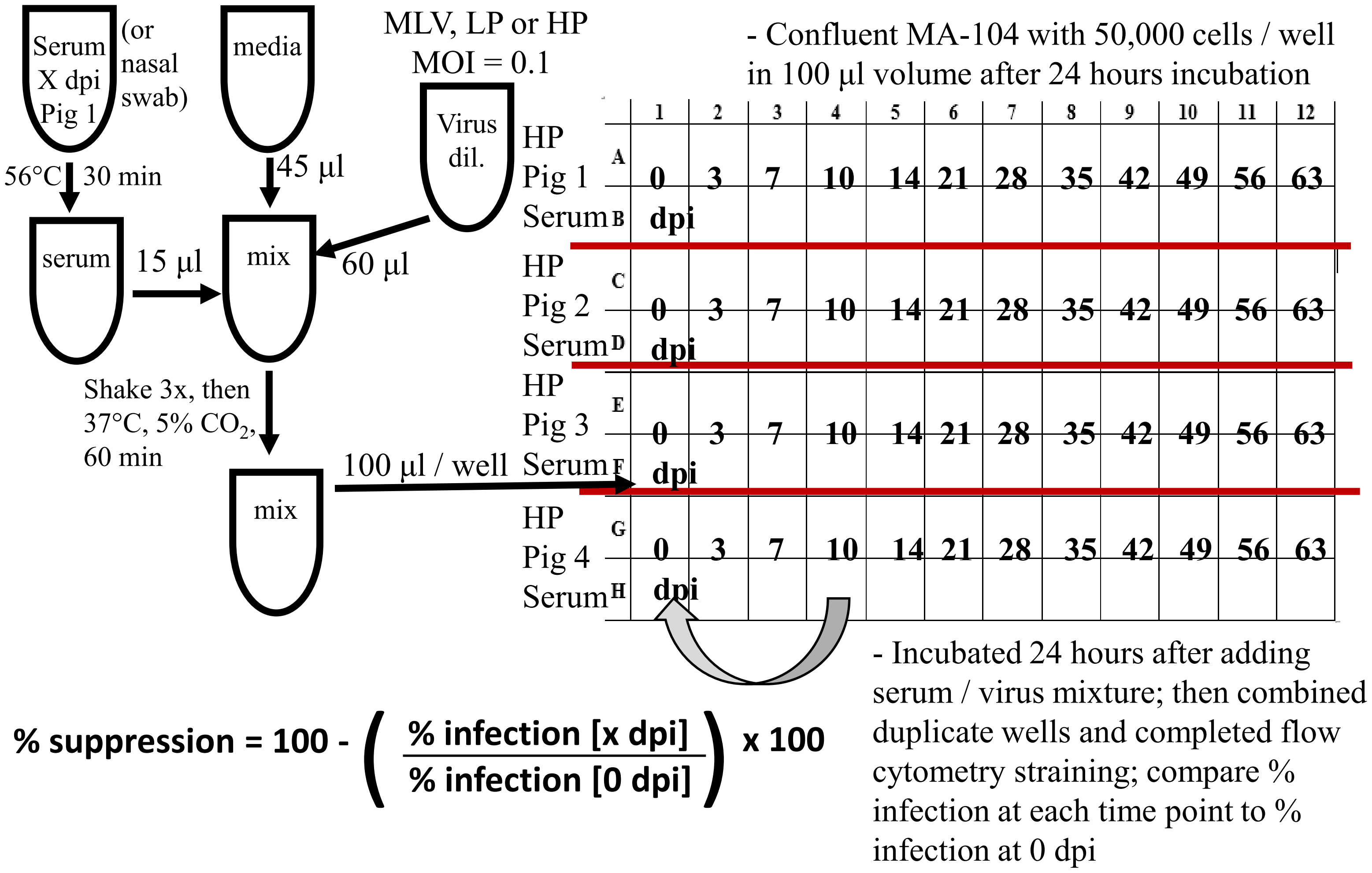

Supplementary Figure 2. Neutralizing antibody quantification using flow cytometry. This graphic illustrates the detailed steps involved in the neutralizing antibody quantification using flow cytometry.
